# Impact of temperature on survival, development and longevity of *Aedes aegypti* and *Aedes albopictus* (Diptera: Culicidae) in Phnom Penh, Cambodia

**DOI:** 10.1186/s13071-025-06892-y

**Published:** 2025-08-27

**Authors:** Bros Doeurk, Sokkeang Leng, Zanory Long, Pierre-Olivier Maquart, Sébastien Boyer

**Affiliations:** 1 Medical and Veterinary Entomology Unit, Institut Pasteur du Cambodge, PO Box 983, Phnom Penh, Cambodia; 2 IRD, UMR-247 Evolution, Génome, Comportement, Ecologie, Université Paris-Saclay, Gif-Sur-Yvette, France; 3 Ecology and Emergence of Arthropod-Borne Diseases, Institut Pasteur, Paris, France

**Keywords:** Arbovirus, Chikungunya virus, Dengue virus, Survival, Longevity, Yellow fever virus, Zika virus

## Abstract

**Background:**

*Aedes aegypti* and *Ae. albopictus* are primary vectors of dengue virus in Cambodia, distributed throughout the country. Climate change is predicted to affect the relative density of these two species, but there is a lack of studies evaluating the impact of temperature on populations of these two species in this region. This study investigates the impact of temperature on the survival, development and longevity of *Ae. aegypti* and *Ae. albopictus* from populations collected in Phnom Penh, Cambodia.

**Methods:**

*Aedes aegypti* and *Ae. albopictus* populations were collected in Phnom Penh. The experiment was conducted in a climatic chamber with temperatures ranging from 15 °C to 40 °C, with a 5 °C increment between each treatment. Bionomic parameters from the F2 egg hatching rate to the number of F3 eggs produced at each temperature treatment were measured.

**Results:**

Temperature significantly influenced all life history traits of *Ae. aegypti* and *Ae. albopictus*. The highest egg hatching rates were observed at 25 °C for *Ae. aegypti* (97.97%) and 20 °C for *Ae. albopictus* (90.63%). Larvae of both species could not survive beyond the first stage at 40 °C. During immature stages, development time decreased at higher temperature (35 °C), but mortality was increased. Female longevity peaked at 25 °C for *Ae. aegypti* (66.7 days) and at 20 °C for *Ae. albopictus* (22.6 days), with males having significantly shorter lifespans. In addition, the optimal temperature for female survival is predicted higher in *Ae. aegypti* than in *Ae. albopictus*, at 27.1 °C and 24.5 °C, respectively. Wing length increased at lower temperatures, with *Ae. aegypti* consistently longer than *Ae. albopictus* at 15 °C and 35 °C. Blood-feeding rates were highest at 30 °C for *Ae. aegypti* (61.0%) and at 25 °C for *Ae. albopictus* (52.5%).

**Conclusion:**

*Aedes albopictus* appears better adapted to lower temperatures, whereas *Ae. aegypti* is better adapted to higher temperatures. Warmer temperatures accelerate mosquito development but also increased mortality and reduced adult longevity, which could influence their ability to transmit pathogens. These findings highlight the critical role of temperature in mosquito biology and emphasize the potential impact of climate change on dengue transmission dynamics in the future.

**Graphical Abstract:**

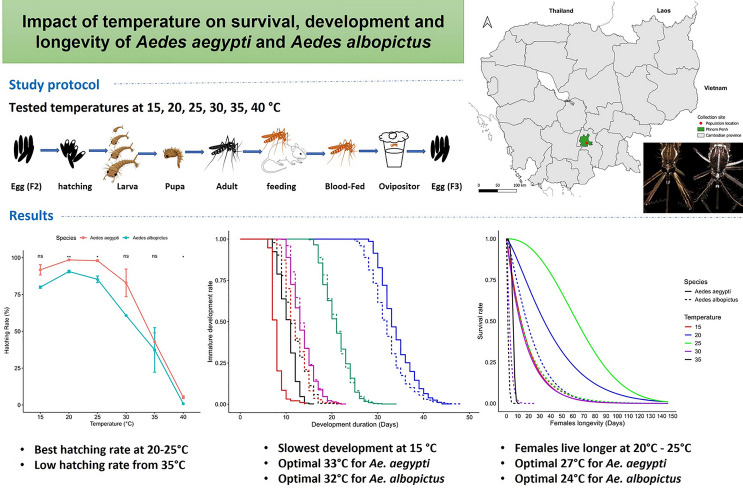

**Supplementary Information:**

The online version contains supplementary material available at 10.1186/s13071-025-06892-y.

## Background

Vector-borne disease is one of the major public health burdens, with approximately 82% of the global population at risk of at least one major vector-borne disease and over half of the world’s population living in areas at risk of two or more major vector-borne diseases [1, 2]. Among the vector-borne diseases, arboviruses are viral pathogens transmitted to humans by arthropod vectors [3, 4]. Mosquitoes are major vectors for arboviruses and are one of the greatest public health concerns worldwide [5]. Among mosquito species, *Aedes aegypti* (Linnaeus, 1762) and *Aedes albopictus* (Skuse, 1894) are identified as highly competent vectors for several major arboviruses worldwide such as dengue virus (DENV), yellow fever virus (YFV), chikungunya virus (CHIKV) and zika virus (ZIKV) [3, 6, 7]. *Aedes albopictus* originated from forested areas in Southeast Asia [7, 8], while *Ae. aegypti* originated from Africa [9]. These two species are widely spread in various ranges of habitats on almost all continents except Antarctica [9]. In addition, both *Ae. aegypti* and *Ae. albopictus* are present in all major Asian cities [9, 10]. In Cambodia, *Ae. aegypti* and *Ae. albopictus* spread across different ecological habitats; the first species prefers urban areas, while the latter is more distributed in wooded and shadier habitats (in the cities) and in forested areas [11]. Both species are recorded from all 25 provinces of Cambodia, in particular at the same locations [11]. Moreover, larvae of these species can be found in various containers, both artificial and natural, and they share the same types of breeding habitats [11].

Global environmental changes will very likely influence the population dynamics of mosquito vectors and the transmission of the pathogens they carry [12–14]. Among the various abiotic factors affected by global environmental changes, temperature is one of the most critical, influencing the development, ecology, distribution, behavior and virus transmission of the two primary dengue vectors, *Ae. aegypti* and *Ae. albopictus* [12–16]. The global distribution of the two species is a consequence not only of global climate changes but also of their different adaptive responses to climate change and ability to diapause [17–20]. *Aedes aegypti* can survive and develop at temperature ranges between 16 ⁰C and 36 ⁰C [21]. Previous studies found that the optimal temperature for *Ae. aegypti* to develop and thrive is between 22 ⁰C and 32 ⁰C, while the longest lifespans of these species are expected to be at around 22 °C [21, 22]. In France, *Ae. albopictus* can fully develop in various temperature ranges between 15 ⁰C and 35 ⁰C in laboratory conditions, although temperatures < 10.4 ⁰C and > 29.7 ⁰C impair their survival and proper development [16]. In mosquitoes, an increase in temperature leads to a decrease in development time and body size [14, 16, 23–25]. However, the adaptation to temperature in both of these species may be region-specific from local selection pressures and has not yet been examined in Southwest Asia.

Based on the model prediction analysis, temperature is defined as one of the key factors impacting the distribution, diversity, behavior and dynamics of the two main dengue vectors, *Ae. aegypti* and *Ae. albopictus*, in Cambodia [11, 19, 26]. Furthermore, the impact of temperature on the biology of these vectors can help us better understand their spatial and temporal distribution, allowing us to assess the risks posed by climate change and design region-specific intervention strategies. Given the public health importance of these two species and the variability observed among their different strains worldwide, it is essential to understand how temperature affects the development, survival and vectorial capacity of these two species to later enable the development of accurate dengue risk prediction models fitted for the country. Therefore, the aim of this research was to conduct a laboratory experiment to investigate the effects of temperature on the biological development and life cycle of *Ae. aegypti* and *Ae. albopictus* populations in Phnom Penh, Cambodia. The experiment assessed various life history traits of both species under controlled temperatures ranging from 15 °C to 40 °C, including hatching success, mortality rates, developmental stages, blood-feeding behavior, egg production, survivorship and adult longevity.

## Methods

### Mosquito collection and maintenance

Field strains of *Ae. aegypti* and *Ae. albopictus* used in this study were collected from the Royal University of Agriculture (RUA), located in Phnom Penh, Cambodia (11.51196 N; 104.9005 E, WGS 84). Mosquito larvae were collected two times, in February 2024 for the higher temperatures (at 40, 35 and 30 °C) and in June 2024 for the lower ones (at 25, 20 and 15 °C) using ovitraps and active searches from surrounding breeding habitats in various containers. All mosquito larvae were transferred to an insectarium at Institut Pasteur du Cambodge (IPC). The F0 adults were identified to the species level following available morphological identification keys [27, 28] and reared separately by species. All adult mosquitoes were maintained under standard rearing conditions at a temperature of 25 ± 2 °C and relative humidity of 75 ± 5% under a photoperiod of 12:12 h (day:night). Adult mosquitoes were fed daily with 10% sucrose solution, except 1 day before blood-feeding to starve the mosquitoes. Then, F1 adults were fed on mouse blood for 45 min twice weekly and kept for oviposition. The F1 eggs were collected from filter paper in plastic cups with de-chlorinated water every day. F1 eggs were kept for a further 3 days at high humidity conditions (RH > 90%) for fully complete embryo development and then dried at 50% relative humidity and stored in envelopes. F1 eggs were stored for 1 to 3 weeks to ensure a sufficient number of eggs, then immersed in the water to produce F1 generation and later amplified to produce eggs for F2 generation following the same protocol. Finally, the F2 eggs were used for our experiment.

### Experimental design

In Cambodia, field temperatures during the active periods of *Aedes* mosquitoes typically range from approximately 24 °C to 35 °C, with occasional extremes reaching up to 40 °C during the hottest months and dropping to around 15 °C in cooler seasons. This study was designed to investigate the impact of temperature on the development of two main dengue vector mosquitoes, *Ae. aegypti* and *Ae. albopictus*, under controlled temperatures of 15, 20, 25, 30, 35 and 40 °C, with a 12:12 h day:night cycle. The selected treatment temperatures were chosen to represent the full range of ecologically relevant temperatures that these populations are likely to encounter in the field. This range also allows us to capture the thermal limits of their development and survival, from suboptimal to potentially lethal temperatures, thus providing a comprehensive understanding of temperature-dependent biological responses. The experiment was conducted in a climatic chamber machine (MT-313 Plant Growth Chamber, Taiwan Hipoint Corporation) with controlled temperature, humidity and LED light. This study was carried out two times, as the machine can set three different temperatures each time. The first experiment was conducted at temperatures of 30, 35 and 40 °C from May to July 2024 and the second at temperatures of 15, 20 and 25 °C from August 2024 to January 2023. Each treatment had three replicates.

### Experimental study

In each replicate, 200 F2 eggs were counted under a stereomicroscope and placed in white plastic trays (32 cm × 22 cm × 4 cm). Each tray was filled with 1 l of dechlorinated water (0.2 larvae/ml) [29] and placed inside the climatic chamber for 24 h before the eggs were put at the designated temperature for each treatment. During the experiment, the trays were randomly repositioned daily to prevent any location effect within the chamber. The hatching rate was assessed 72 h (3 days) after exposure to temperatures of 40 °C, 35 °C and 30 °C and 120 h (5 days) after exposure to 25 °C, 20 °C and 15 °C to allow sufficient time for egg hatching at lower temperatures. Mosquito larvae were fed daily with 0.3 mg/larva/day [29] crushed rabbit food (Rabbit 9999, Thailand). Pupae were counted daily and transferred to breeding containers within the chamber until they emerged as adults. Then, newly emerged adults from each replicate were counted daily, transferred to a single adult cage (21 cm × 21 cm × 21 cm) and provided with 10% sucrose solution daily. Adult mosquitoes were maintained for 10 days from the first emergence to ensure an adequate number of female mosquitoes for mating. Sugar was removed 24 h before blood-feeding to starve the female mosquitoes. The mosquitoes were then blood-fed on mice for 45 min. Each time, a maximum of five blood-fed females were randomly selected from each cage and placed individually on filter paper inside a plastic cup covered with a net for oviposition. After 5 days, the number of F3 eggs laid per female was recorded, and the female mosquitoes from each cup were freeze-killed for wing measurements [23]. The remaining adult mosquitoes in the cages were monitored daily to count the number of dead mosquitoes until all individuals had died.

### Adult body size

A total of 30 dead females from each treatment were randomly selected for body size measurements of each species at each temperature. The right wings of female mosquitoes were dissected and placed on glass slides. All wings were measured from the apical notch to the axillary margin using a micrometer [30].

### Statistical analysis

We tested two mosquito species (*Ae. aegypti* and *Ae. albopictus*) at six constant temperatures (15, 20, 25, 30, 35 and 40 °C). For each temperature (six) and species (two) combination, three independent replicates were conducted, resulting in 36 experimental units. The measured response variables included hatching rate, survival rate, mortality rate, feeding rate, development rate, adult emergence, egg production, body size and adult longevity.

The normality of the data was tested using a Shapiro-Wilk test before selecting the appropriate statistical tests. Two-way ANOVA was used to test the effects of temperature and species as well as their interactions on egg hatching, survival of immature stages, adult emergence, blood-feeding, wing length and number of eggs laid for each species across different temperatures. One-way ANOVA was used to test the effect of temperature on each species, while Tukey HSD tests were used for post hoc pairwise comparisons after significance had been detected in each analysis. Student’s t-test was also used to compare the differences between the two species. The duration development for the immature stage and adult longevity of both species was modeled using the Weibull model [31] for each species and temperature. We used the *fitdist* function from the *fitdistrplu*s package to fit the Weibull distribution. This model provided estimated times (probabilities) by summarizing the mean, median and maximum duration for each species and temperature condition.

To determine the optimal, lower and upper temperatures for the development time of the immature stage in each species, the development rate was first calculated as the reciprocal of the development time (1/day), where development time represents the number of days from hatching to adult emergence. To model the relationship between temperature and development rate, we fitted the Logan-10 nonlinear thermal performance model [32], defined as

$$Dr\, = \,a\,e^{bt} \,(T_{\max} \, - T)$$

where *D*_*r*_ is the development rate, *T* is the rearing temperature (°C), *T*_max_ is the upper critical temperature, and *a* and *b* are fitted parameters. The model was fitted separately for each species using nonlinear least squares regression (*nlsLM*) in R. Model parameters were estimated with starting values (*a* = 0.0001, *b* = 0.1, *T*_*max*_ = 40) and constrained within tested ranking temperature. The lower (*T*_min_​) and upper (*T*_max_​) thermal limits were determined based on model predictions, approximating the lower and upper threshold. The optimal temperature (*T*_opt_) was approximated as

$$T_{opt} \, = \,T_{\max } \, - \,1/b$$

To assess the relationship between temperature and female survival, we aggregated the total number of surviving females per day for each temperature and species. We then fitted a quadratic regression model to estimate the optimal temperature (*T*_opt_​), lower threshold (*T*_min_​) and upper threshold (*T*_max_​) for survival [33]. The model was defined as:

Quadratic model = *β*_0_ + *β*_1_(Temperature) + *β*_2_(Temperature^2^) + *ϵ.*

Here, *β*_0_​, *β*_1_​ and *β*_2_​ are regression coefficients, and *ϵ* is the residual error. The lower (*T*_min_​) and upper (*T*_max_) thermal limits were estimated as the temperatures at which the predicted survival approached zero. The optimal temperature (*T*_opt_​) was calculated as:

$$T_{{{\text{opt}}}} = \, - \beta {1 }/{ 2}\beta_{{2}}$$

All statistical analyses were performed using R software version 4.2.0, and significance was accepted at a *P*-value < 0.05.

## Results

### The egg hatching rate

**Fig. 1:**
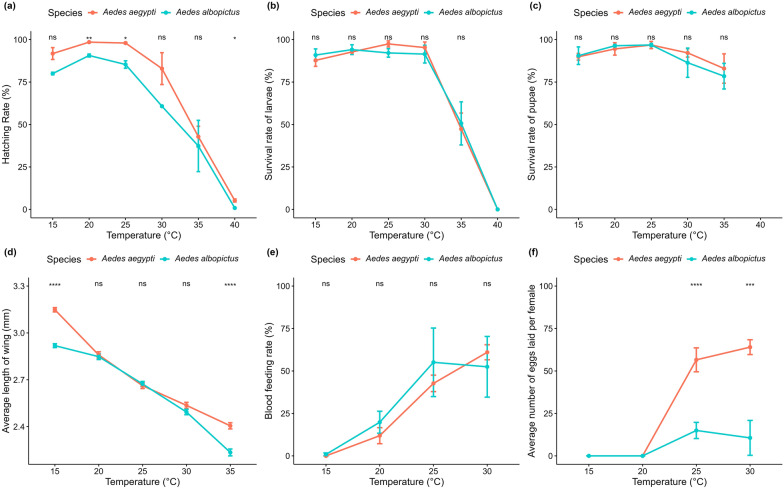
**a** Hatching success, **b** larval survival rate, **c** pupal survival rate, **d** wing length, **e** blood-feeding rate, **f** egg-laying of female *Aedes aegypti* and *Aedes albopictus* mosquitoes at different temperatures. Lines represent mean ± standard error (SE). Statistical comparisons between species at each temperature were performed using Student’s t-test with significance accepted at ns: *p* > 0.05 (ns), *p < 0.05, **p < 0.01, ***p < 0.001 and ****p < 0.0001

Eggs of *Ae. aegypti* and *Ae. albopictus* hatched across all tested temperatures ranging from 15 °C to 40 °C (Fig. 1a**; **Table 1). Hatching rates were significantly influenced by temperature (two-way ANOVA, *F*_(5,23)_ = 80.65, *P* < 0.0001) and species (two-way ANOVA, *F*_(1,23)_ = 9.530, *P* = 0.005), but no significant interaction was observed between these factors (two-way ANOVA, *F*_(5,23)_ = 0.566, *P* > 0.05) (Additional file 3: Table S1). The highest hatching rates were recorded at 20 °C, with an average of 98.50 ± 0.29% for *Ae. aegypti* and 90.63 ± 0.88% for *Ae. albopictus* (Table 1). In contrast, the lowest rates were observed at 40 °C, averaging 5.26 ± 0.92% for *Ae. aegypti* and < 1% (0.83 ± 0.33%) for *Ae. albopictus*. We also found that *Ae. aegypti* had significantly higher hatching rates than *Ae. albopictus* at 20 °C, 25 °C and 40 °C (t-test, *t* = 4.51–8.52, *df* = 2, *P* < 0.05), whereas no significant differences were observed at 15 °C, 30 °C and 35 °C (Fig 1a t-test, *t* = 0.33–3.29, *df* = 2, *P* > 0.05). Statistical analysis revealed a significantly higher hatching rate at lower (15–30 °C) than higher temperatures (35 and 40 °C) for both *Ae. aegypti* (ANOVA, *F*_(5,12)_ = 61.92, *P* < 0.0001) and *Ae. albopictus* (ANOVA, *F*_(5,11)_ = 28.09, *P* < 0.0001), and no significant differences were observed among 15, 20, 25 and 30 °C.

**Table 1:** Hatching success, larval survival rate, pupal survival rate, blood-feeding rate, egg-laying rate of female mosquitoes, wing length of *Aedes aegypti* and *Aedes albopictus* under different temperatures. Data are presented as mean ± standard error (SE)

Species	Temperature (°C)	Hatching rate (%) (mean ± SE)	Survival of larvae (%) (mean ± SE)	Survival of pupae (%) (mean ± SE)	Blood-feeding (%) (mean ± SE)	Egg laid (n) (mean ± SE)	Wing (mm) (mean ± SE)
*Ae. aegypti*	40	5.26 ± 0.92^a^	0.00 ± 0.00^a^	–	–	–	–
	35	42.82 ± 6.12^b^	47.32 ± 9.41^b^	82.98 ± 8.66^a^	–	–	2.41 ± 0.02^a^
	30	82.92 ± 9.38^c^	95.31 ± 3.26^c^	92.17 ± 2.47^ab^	61.02 ± 4.43^a^	64.03 ± 4.37^a^	2.54 ± 0.02^b^
	25	97.97 ± 0.52^c^	97.45 ± 1.78^c^	96.82 ± 2.13^b^	42.69 ± 4.84^b^	56.57 ± 7.05^a^	2.66 ± 0.02^c^
	20	98.50 ± 0.29^c^	92.72 ± 0.15^c^	93.78 ± 3.21^b^	11.89 ± 4.72^c^	0.00 ± 0.00^b^	2.86 ± 0.02^d^
	15	91.78 ± 3.49^c^	87.78 ± 3.53^c^	89.89 ± 1.97^ab^	0.00 ± 0.00^c^	–	3.15 ± 0.01^e^
*Ae. albopictus*	40	0.83 ± 0.33^a^	0.00 ± 0.00^a^	–	–	–	–
	35	37.35 ± 15.11^b^	50.67 ± 12.68^b^	78.45 ± 7.54^a^	–	–	2.24 ± 0.02^a^
	30	60.87 ± 0.35^c^	91.45 ± 5.24^c^	86.39 ± 8.61^ab^	52.46 ± 17.88^a^	10.60 ± 10.27^ab^	2.50 ± 0.02^b^
	25	85.32 ± 2.19^c^	92.15 ± 2.53^c^	96.84 ± 0.95^b^	55.11 ± 20.19^a^	14.96 ± 4.76^a^	2.67 ± 0.02^c^
	20	90.63 ± 0.88^c^	94.09 ± 2.88^c^	96.38 ± 0.80^b^	19.81 ± 6.48^ab^	0.00 ± 0.00^ab^	2.85 ± 0.02^d^
	15	80.00 ± 0.76^c^	90.88 ± 3.64^c^	90.52 ± 5.18^ab^	0.88 ± 0.88^b^	0.00 ± 0.00^a^	2.92 ± 0.01^e^

Different superscript letters within each species and parameter indicate significant differences among temperatures (P < 0.05)

### Larval survival rate

Temperature significantly influenced the survival rate of larvae (two-way ANOVA, *F*_(5,23)_ = 108.07, *P* < 0.0001), but no significant differences were found between species (Fig. 1b, two-way ANOVA, *F*_(1,23)_ = 0.001, *P* > 0.05), and there was no interaction between these factors (two-way ANOVA, *F*_(5,23)_ = 0.230, *P* > 0.05) (Additional file 3: Table S1). Larval survival exceeded > 87% for both species at 15, 20, 25 and 30 °C (Fig. 1b; Table 1). The highest larval survival rate was observed at 25 °C for *Ae. aegypti* (97.45 ± 1.78%) and 20 °C for *Ae. albopictus* (94.09 ± 2.88%). In contrast, the lowest larval survival rate was observed at 40 °C for both species, where larvae failed to survive beyond the first instar. In addition, our results revealed a significantly higher larval survival rate at lower temperatures (15–30 °C) compared to the two highest temperature treatments (35 and 40 °C) for both *Ae. aegypti* (ANOVA, *F*_(5,12)_ = 79.89, *P* < 0.0001) and *Ae. albopictus* (ANOVA, *F*_(5,11)_ = 39.33, *P* < 0.0001), and no significant differences were observed among 15, 20, 25 and 30 °C.

### Pupal survival rate

Pupal survival rate was significantly influenced by temperature (two-way ANOVA, *F*_(4,19)_ = 3.58, *P* = 0.025), but no significant differences were found between the species (Fig. 1c, two-way ANOVA, *F*_(1,19)_ = 0.2, *P* > 0.05) and no interaction between the temperature and species (two-way ANOVA, *F*_(4,19)_ = 0.23, *P* > 0.05) (Additional file 3: Table S1). The highest pupal survival was recorded at 25 °C for both species, with 96.82 ± 2.13% for *Ae. aegypti* and 96.84 ± 0.95% for *Ae. albopictus*. In contrast, the lowest survival rate was observed at 35 °C, with 82.98 ± 8.66% for *Ae. aegypti* and 78.45 ± 7.54% for *Ae. albopictus* (Fig. 1c**; **Table 1). Statistical analysis indicated a significantly higher pupal survival rate for both species at 20 and 25 °C compared to 35 °C (ANOVA, *F*_(4,23)_ = 4.27, *P* = 0.01).

### Immature development duration

**Fig. 2:**
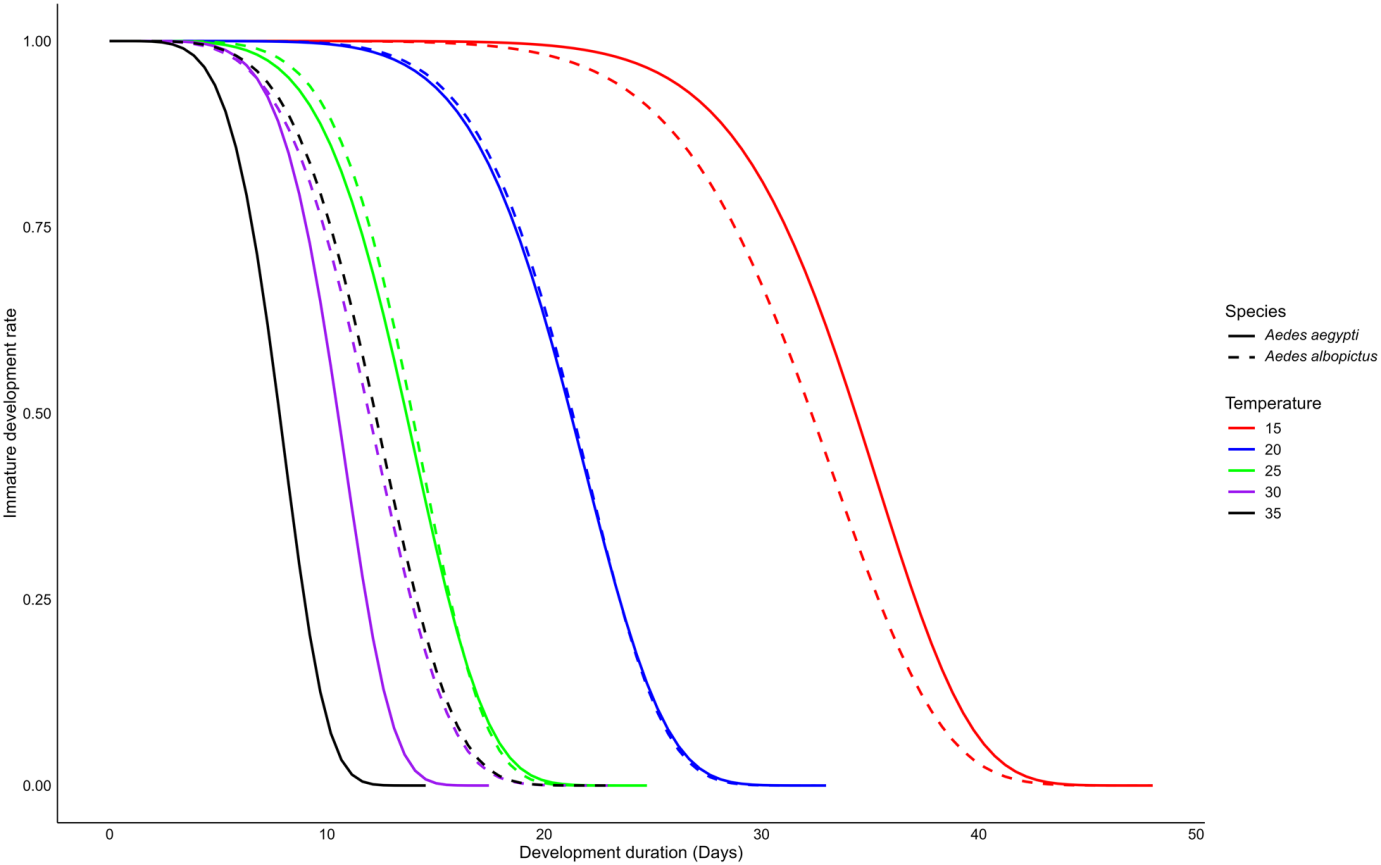
Developmental duration of immature stages of *Aedes aegypti* and *Aedes albopictus* across different temperatures

The development duration of immature stages (from egg to adult emergence) varied significantly across the tested temperature range (15–35 °C) (two-way ANOVA, *F*_(5,23)_ = 96.6, *P* < 0.0001) and between species (two-way ANOVA, *F*_(1,23)_ = 6.42, *P* = 0.01), and significant interaction was observed between these factors (two-way ANOVA, _*F(*4,19)_ = 9.93, *P* = 0.0001). Weibull’s model highlighted that the shortest development duration for *Ae. aegypti* occurred at 35 °C (7.83 days), while *Ae. albopictus* exhibited its shortest development time at 30 °C (11.80 days). In contrast, the development duration decreased with increasing temperature, with the longest durations observed at 15 °C, with 34 days for *Ae. aegypti* and 32 days for *Ae. albopictus*. Interestingly, some individuals in the final immature stages took longer at 15 °C, with *Ae. aegypti* spending up to 46 days and *Ae. albopictus* up to 44 days (Fig. 2**, **Table 2). We also found no statistical difference between the two species at all tested temperatures (t-test, *t* = – 2.84–1.82, df = 2–3, *P* > 0.05), except at 35 °C, where *Ae. aegypti* developed significantly faster than *Ae. albopictus* (t-test, *t* = – 5.33, df = 2, *P* = 0.02), with 7.83 days and 12.2 days, respectively.

**Table 2:** Developmental duration of immature stage and longevity of adult mosquitoes. Data are presented as mean – maximum (day)

Species	Temperature (°C)	Immature stage (day) (Mean – maximum)	Larvae (day) (Mean – maximum)	Pupa (day) (Mean – maximum)	Adult longevity (day) (Mean – maximum)	Female longevity (day) (Mean – maximum)	Male longevity (day) (Mean – maximum)
*Ae. aegypti*	40	–	–	–	–	–	–
	35	7.83–14^a^	6.30–12^a^	1.97–7^a^	5.99–8^a^	5.86–8^a^	6.14–9^a^
	30	10.40–16^b^	8.98–14^b^	1.80–5^a^	15.6–43^a^	17.3–43^a^	14.2–36^ab^
	25	13.50–23^c^	11.10–21^c^	2.68–5^b^	43.2–123^a^	66.7–122^b^	23.6–108^b^
	20	21.00–34^d^	16.6–29^d^	4.69–14^c^	29.7–146^b^	41.3–146^c^	20.5–100^ab^
	15	34.00–46^e^	25.5–38^e^	9.29–14^d^	13.3–78^c^	18.0–78^a^	8.41–76^ab^
*Ae. albopictus*	40	–	–	–	–	–	–
	35	12.2–22^a^	10.7–20^a^	2.01–13^a^	1.92–11^a^	1.41–8^a^	2.46–13^a^
	30	11.80–21^a^	9.92–21^a^	2.65–9^a^	3.70–23^b^	3.15–23^a^	4.35–8^a^
	25	13.8–22^b^	11.00–19^b^	2.97–6^a^	14.3–88^b^	19.0–87^b^	9.43–63^a^
	20	21.1–31^c^	16.9–27^c^	4.46–8^a^	14.5–96^b^	22.6–99^ab^	7.42–40^a^
	15	32.1–48^d^	23.5–44^d^	9.35–21^b^	13.1–71^b^	18.9–70^a^	7.45–72^a^

Different superscript letters within each species and parameter indicate significant differences among temperatures (P < 0.05)

Differences in the duration of immature development were attributed to varying rates of progression through each immature stage, including egg hatching, larval development and pupation. During larval stage, the development duration was significantly influenced by temperature (two-way ANOVA, *F*_(4,19)_ = 509.98, *P* < 0.0001) between the two species (two-way ANOVA, *F*_(1,19)_ = 5.74, *P* < 0.0001, *P* > 0.05). A significant interaction between the temperature and species was also observed (two-way ANOVA, *F*_(4,19)_ = 13.94, *P* < 0.0001). The longest larval development duration was recorded at 15 °C for both species, averaging 25.5 days for *Ae. aegypti* and 23.5 days for *Ae. albopictus*. Additionally, the last individual larvae took the longest to develop at 15 °C, with a maximum duration of 38 days for *Ae. aegypti* and 44 days for *Ae. albopictus* (Additional file 1: Fig. S1a). In contrast, the shortest larval development durations were observed at 35 °C for *Ae. aegypti* and 30 °C (9.92 days) for *Ae. albopictus*. In particular, *Ae. aegypti* larvae developed signifyingly faster than *Ae. albopictus* at 30 (t-test, *t* = – 5.98, df = 2, *P* = 0.01) and 35 °C (t-test, *t* = – 8.03, df = 2, *P* = 0.009), while no significant differences were observed at 15, 20 and 25 °C (Additional file 1: Fig. S1b, Table 2, t-test, *t* = – 0.97–2.06, df = 2, *P* > 0.05).

During pupal stage, the development duration was significantly influenced by temperature (two-way ANOVA, *F*_(4,19)_ = 98.91, *P* < 0.0001), but no significant differences were found between species (two-way ANOVA, *F*_(1,19)_ = 2.26, *P* > 0.05), and there was no interaction between these factors (two-way ANOVA, *F*_(4,19)_ = 0.8, *P* > 0.05). Similar to larvae, the longest duration of pupal development duration was recorded at 15 °C for both species, averaging 9.29 (maximum 14) days for *Ae. aegypti* and 9.35 (maximum 21) days for *Ae. albopictus*. In contrast, the fastest development duration was recorded at 35 °C for both species, averaging 1.97 (maximum 7) days for *Ae. aegypti* and 2.01 days for *Ae. albopictus* (maximum 13 days) (Table 2, Additional file 1: Fig. S1c). However, no significant differences were observed between the two species at any temperature (Table 2, Additional file 1: Fig. S1d, t-test, *t* = – 2.52–0.67, df = 1–3, *P* > 0.05).

### Adult male and female longevity

**Fig. 3:**
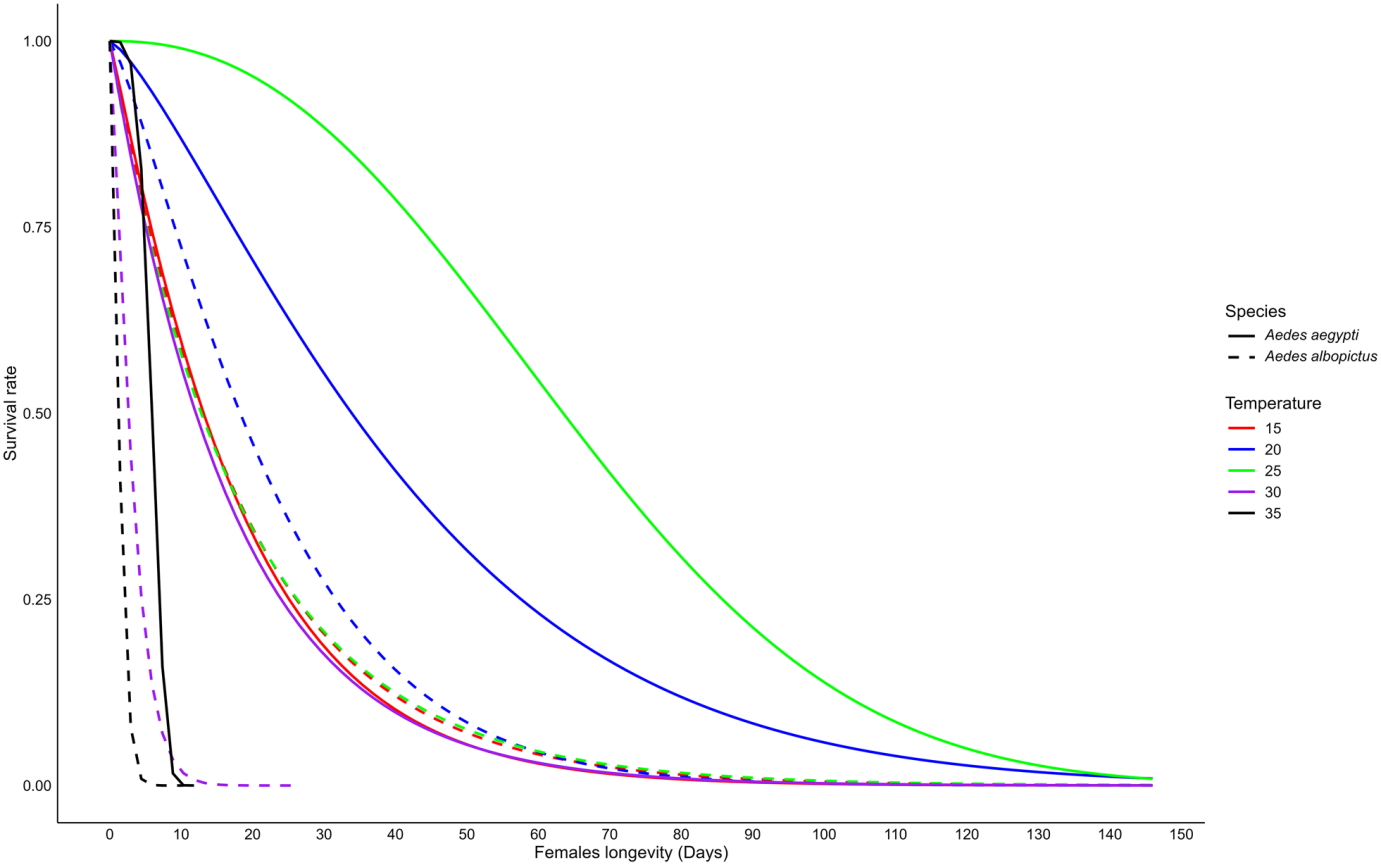
Longevity of female *Aedes aegypti* and *Aedes albopictus* mosquitoes reared at different temperatures

We found that female longevity for each species was significantly strongly affected by temperature (two-way ANOVA, *F*_(4,19)_ = 34.91, *P* < 0.0001) and species (two-way ANOVA, *F*_(1,19)_ = 48.08, *P* < 0.0001). Additionally, a significant interaction between temperature and species was observed (two-way ANOVA, *F*_(4,19)_ = 12.08, *P* < 0.0001). Weibull's model estimated that *Ae. aegypti* females had the longest survival at 25 °C, with an average lifespan of 66.7 days and a maximum of 122 days. However, the last surviving *Ae. aegypti* female lived up to 146 days at 20 °C. Regarding *Ae. albopictus* females, the longest survival was observed at 20 °C, with an average lifespan of 22.6 days and a maximum of 99 days. In contrast, the shortest female lifespan was recorded at 35 °C for both species, averaging 5.86 (maximum 8) days for *Ae. aegypti* and 1.41 (maximum 8) days for *Ae. albopictus* (Fig. 3; Table 2**, **Additional file 2: Fig. S2a). We also found that female longevity was significantly longer at 20 °C and 25 °C compared to 15 °C, 30 °C and 35 °C for both *Ae. aegypti* (ANOVA, *F*_(4,10)_ = 41.53, *P* < 0.0001) and *Ae. albopictus* (ANOVA, *F*_(4,9)_ = 41.53, *P* = 0.01). Between the two species, *Ae. aegypti* females lived significantly longer than *Ae. albopictus* females at 25, 30 and 35 °C (t-test, *t* = 5.42–12.4, df = 2–3, *P* < 0.05). However, no significant difference was observed at 15 °C and 20 °C (Additional file 2: Fig. S2b, t-test, *t* = −0.02–2.40, df = 2, *P* > 0.05).

**Fig. 4:**
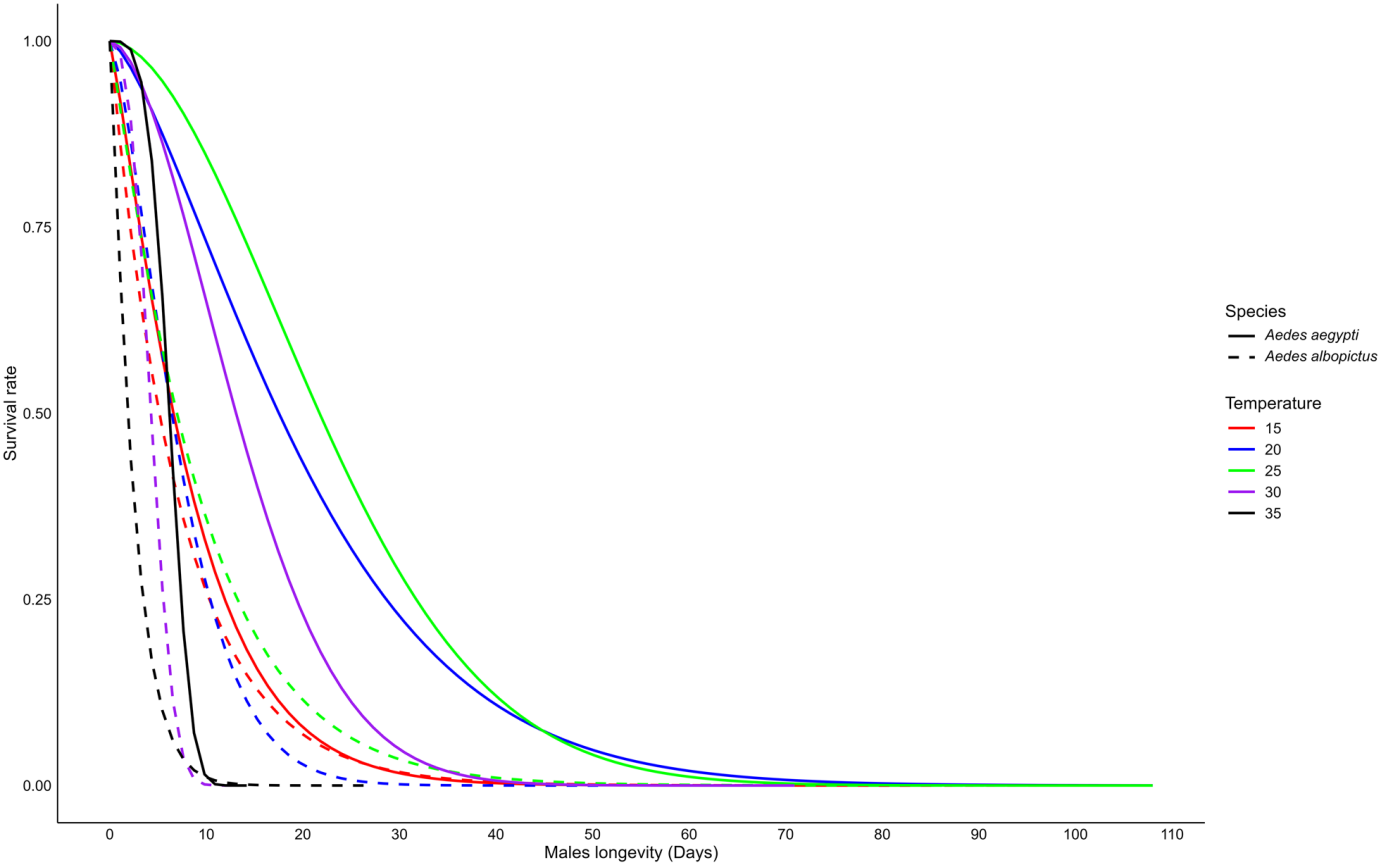
Longevity of male *Aedes aegypti* and *Aedes albopictus* mosquitoes reared at different temperatures

Our results show a significant difference in longevity between male and female mosquitoes, with females having notably longer lifespans for both *Ae. aegypti* species (t-test, *t* = 2.39, df = 16, *P* = 0.02) and *Ae. albopictus* (t-test, *t* = 2.38, df = 16, *P* = 0.03). We found that male longevity for each species was significantly influenced by temperature (two-way ANOVA, *F*_(4,19)_ = 12.32, *P* < 0.0001) and species (two-way ANOVA, *F*_(1,19)_ = 47.24, *P* < 0.0001). Additionally, a significant interaction between temperature and species was observed (two-way ANOVA, *F*_(4,19)_ = 5.00, *P* = 0.006). Weibull's model estimated the longest surviving of males was at 25 °C for both species, averaging 23.6 (maximum 108) days for *Ae. aegypti* and 9.43 (maximum 63) days for *Ae. albopictus*. In contrast, the shortest male lifespan was recorded at 35 °C for both species, averaging 6.14 (maximum 9) days for *Ae. aegypti* and 2.46 (maximum 13) days for *Ae. albopictus* (Fig. 4**; **Table 2**, **Additional file 2: Fig. S2c). We also found that male longevity was significantly longer at 20 °C and 25 °C compared to 15 °C, 30 °C and 35 °C for *Ae. aegypti* (ANOVA, *F*_(4,10)_ = 31.27, *P* < 0.0001), but no significant difference was recorded for *Ae. albopictus* (ANOVA, *F*_(4,9)_ = 0.85, *P* > 0.05). Between the two species, *Ae. aegypti* males lived significantly longer than *Ae. albopictus* females at 20, 25 and 30 °C (t-test, *t* = 5.45–9.07, df = 2–3, *P* < 0.05). However, no significant difference was observed at 15 °C and 35 °C (Additional file 2: Fig. S2d, t-test, *t* = 0.44–1.16, df = 2, *P* > 0.05).

### Temperature thresholds

**Fig. 5:**
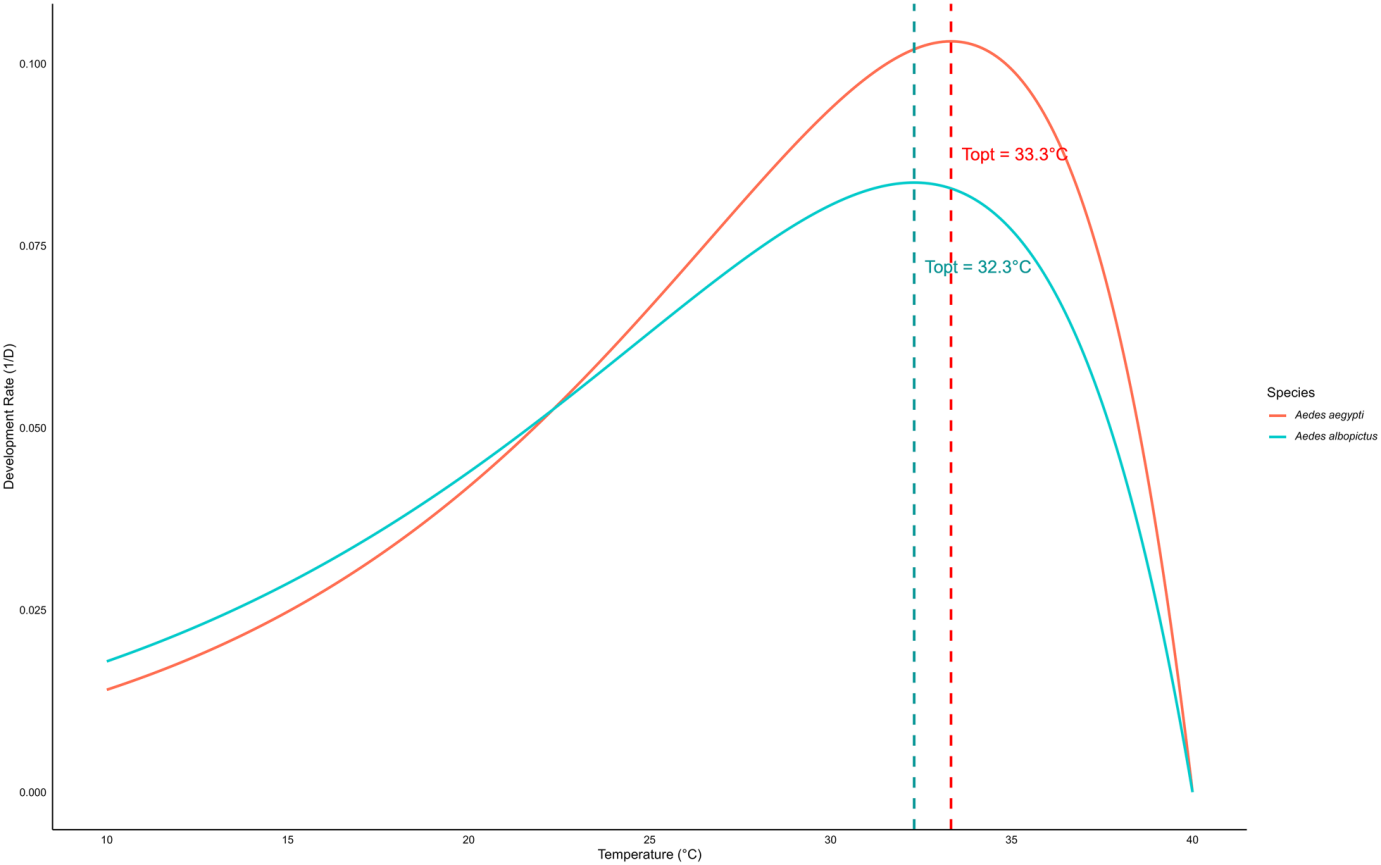
Estimated optimal, upper and lower temperature thresholds for development rates of immature stages in *Aedes aegypti* and *Aedes albopictus*. The optimal temperature indicates the peak development rate, and the lower and upper thresholds represent the temperature limits where the development rate drops to zero

**Fig. 6:**
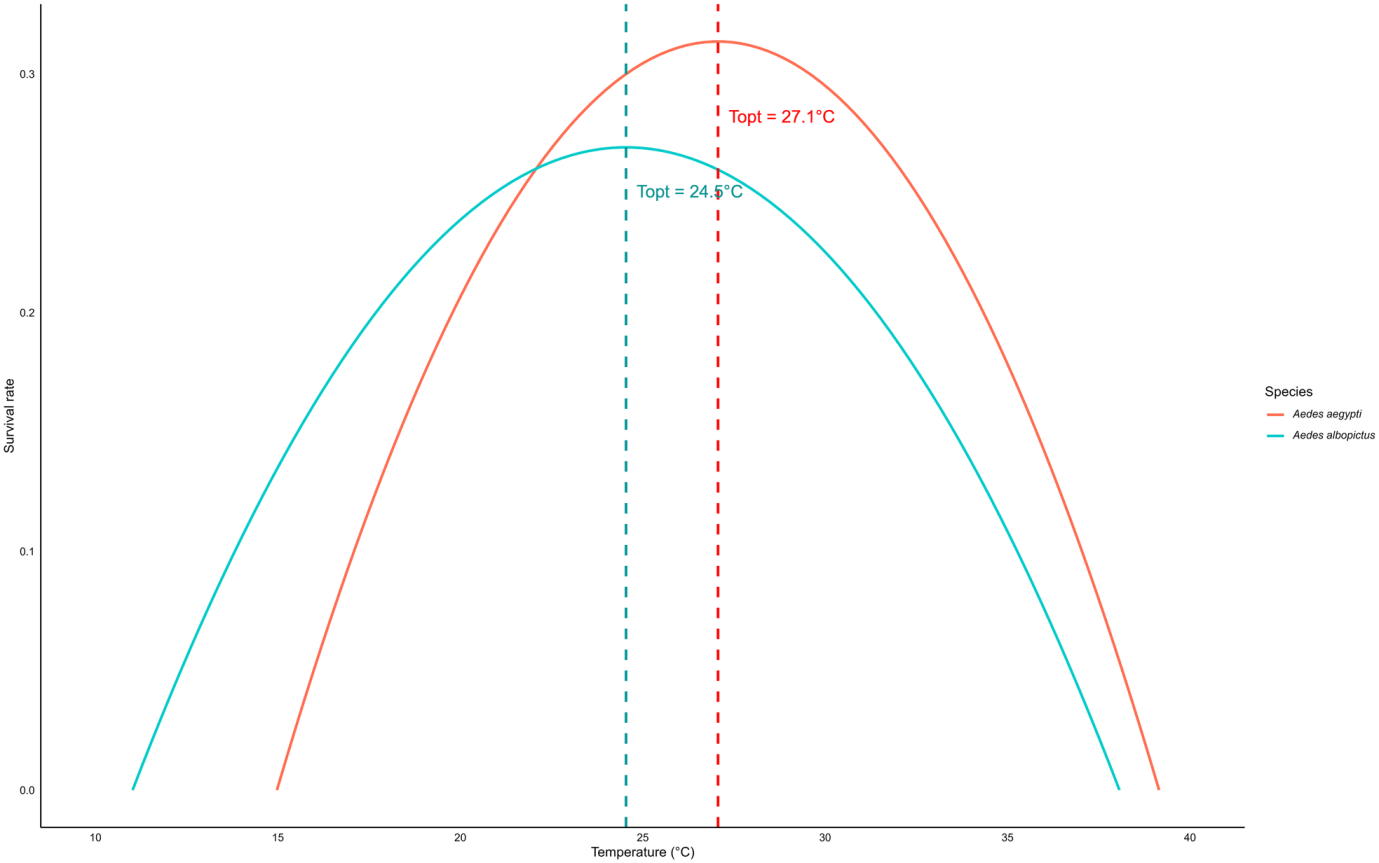
Estimated optimal, upper and lower temperatures for survival of *Aedes aegypti* and *Aedes albopictus* females. The optimal temperature indicates the peak survival rate, and the lower and upper thresholds represent the temperature limits where the survival rate drops to zero

The development rate during the immature stage of *Ae. aegypti* and *Ae. albopictus* followed a non-linear pattern with temperature, best described by the Logan regression model (Fig. 5). The estimated optimal temperature (*T*_opt_) was 33.33 °C for *Ae. aegypti* and 32.31 °C for *Ae. albopictus*, with development rates declining at both lower and higher temperatures. The upper thermal limit (*T*_max_​) was 40.0 °C for both species, while the lower threshold (*T*_min_) was approximated at 5 °C (Fig. 5). The survival of female *Ae. aegypti* and *Ae. albopictus* followed a quadratic trend model with temperature (Fig. 6). The estimated optimal temperature (*T*_opt_​) was 27.06 °C for *Ae. aegypti* and 24.54 °C for *Ae. albopictus*, with survival declining at both lower and higher temperatures. The lower (*T*_min_) and upper (*T*_max_​) thermal limits were 14.97–39.15 °C for *Ae. aegypti* and 11.02–38.07 °C for *Ae. albopictus* (Fig. 6).

### Body size of female mosquitoes

Body size of mosquitoes was indicated by the length of the female's wings for each species. The wing length of *Ae. aegypti* and *Ae. albopictus* varied significantly across the tested temperatures (two-way ANOVA, *F*_(4,750)_ = 404.35, *P* < 0.0001) and between species (two-way ANOVA, *F*_(1,750)_ = 49.37, *P* < 0.0001); significant interaction was also observed between these factors (Fig. 1d**; **Table 1, two-way ANOVA, _*F(*4,750)_ = 18.55, *P* < 0.0001) (Additional file 3: Table S1). A clear trend was observed, with wing length increasing as the temperature decreased. For *Ae. aegypti*, the longest average wing length was observed at 15 °C (3.15 ± 0.01 mm), while the shortest was at 35 °C (2.42 ± 0.02 mm). Similarly, for *Ae. albopictus*, the longest average wing length occurred at 15 °C (2.92 ± 0.01 mm), and the shortest was at 35 °C (2.24 ± 0.02 mm) (Fig. 1c; Table 1). Statistical analysis revealed *Ae. aegypti* had significantly longer wings than *Ae. albopictus* at 15 °C and 35 °C (t-test, *t* = 5.45–12.4, df = 67–177, *P* < 0.05), but no significant difference was observed between the two species at 20 °C, 25 °C and 30 °C (t-test, *t* = – 0.44–1.66, df = 136–177, *P* > 0.05).

### Blood-feeding rate

Blood-feeding rates of female mosquitoes were assessed during two feeding trials conducted at temperatures ranging from 15 °C to 30 °C, excluding 35 °C because of high adult mortality before the feeding day. The average number of blood-fed females was significantly influenced by temperature (two-way ANOVA, *F*_(3,37)_ = 19.56, *P* < 0.0001), but no significant differences were found between the species (Fig. 1d, two-way ANOVA, *F*_(1,37)_ = 0.34, *P* > 0.05) and no interaction between the species (two-way ANOVA, *F*_(3,37)_ = 0.52, *P* > 0.05) (Additional file 3: Table S1). The highest feeding rate was observed at 30 °C for *Ae. aegypti* (61.02 ± 4.43%) and 25 °C for *Ae. albopictus* (52.46 ± 17.88%) (Fig. 1e; Table 1). In contrast, the lowest blood-feeding rates were recorded at 15 °C for both species, where *Ae. aegypti* females did not feed at all and only three *Ae. albopictus* individuals fed (0.88 ± 0.88%). We also found that the blood-feeding rate of both species was not significantly different at all tested temperatures (15–35 °C) (t-test, *t* = – 0.59–0.46, df = 3–9, *P* > 0.05).

### Number of eggs laid

Our results indicate that the average number of eggs laid per female mosquito varied significantly across the tested temperature range (15–30 °C) (two-way ANOVA, *F*_(3,156)_ = 42.39, *P* < 0.0001) and between the two species (two-way ANOVA, *F*_(1,156)_ = 49.02, *P* < 0.0001), and there was a significant interaction between these factors (two-way ANOVA, *F*_(2,156)_ = 16.95, *P* < 0.0001) (Additional file 3: Table S1). The highest egg production was recorded at 30 °C for *Ae. aegypti*, with an average of 64.03 ± 4.37 eggs per female, and at 25 °C for *Ae. albopictus*, with an average of 14.96 ± 4.76 eggs per female. In contract, the lowest egg reproduction was recorded at 15 °C, where three *Ae. albopictus* individuals that fed on blood did not lay any eggs (0.00 ± 0.00 eggs/female). For *Ae. aegypti*, egg production at 15 °C was unknown, as no females fed on blood at this temperature. We also found that *Ae. aegypti* females produced significantly more eggs than *Ae. albopictus* at 25 and 30 °C (t-test, *t* = 4.89–4.79, df = 12–50, *P* < 0.05) (Fig. 1f; Table 1).

## Discussion

### Biology: hatching, development, survival and mortality

Temperature plays a crucial role in shaping the development, survival, longevity, feeding behavior and reproduction of *Ae. aegypti* and *Ae. albopictus*, which could affect their ability to transmit arboviruses. In our study, despite differences in overall survival rates, both species completed their life cycle between 15 °C and 35 °C. However, at temperatures > 40 °C, the lowest hatching rates (< 6%) were observed and all larvae failed to survive beyond the first instar. This highlights an upper thermal limit of their larval development. Despite this sharp decline, few larvae were still able to hatch at 40 °C [34–36], suggesting that both species could potentially survive under thermal stress. This is relevant in the context of ecology, as both species prefer to breed in a variety artificial and natural containers [11]. Indeed, shaded or water-retaining artificial containers, such as used tires, flower pots and water storage tanks, can serve as microhabitats that buffer extreme temperatures, allowing eggs to remain viable even in hot environments. Thermal stress may constrain egg survival under extreme conditions, and such limitations likely vary depending on mosquito strain and the specific climatic conditions to which they are adapted [37].

Immature stages for both species exhibited higher survival at lower temperatures between 15 °C and 30 °C, but survival declined at the two highest temperature treatments, particularly at 35 °C and 40 °C. Previous studies have showed that temperatures between 15 °C and 28 °C are more favorable for mosquito development, while higher temperatures impose physiological stress, increasing mortality [15, 38, 39]. Increasing temperatures accelerate mosquito development by increasing metabolic rates, leading to faster larval growth and shorter life cycles and increasing the gonotrophic cycles [13, 40]. Consequently, the fast development of immature stages was recorded between 32 and 33 °C for both species (optimal temperature for immature development rates), potentially increasing their population growth rate, but this advantage is counterbalanced by significantly higher mortality rates at higher temperatures. These findings support the general observation that, as poïkilotherms, metabolic rates of insects increase with temperature, which can become counterproductive and ultimately lethal at higher temperatures [41–44]. In addition to temperature effects, differences in hatching success and speed between *Ae. aegypti* and *Ae. albopictus* across the temperature treatments may have influenced immature survival outcomes. Indeed, as a low hatching rate led to a lower density of larvae, the consequent variations in larval density could have affected competition for food and space, potentially influencing mortality rates under certain thermal conditions [45, 46].

While our results align with previous observations that describe 20–30 °C as the favorable temperature range for immature stage survival in these species [24, 47–49], we found that survival was generally high across the 15 °C to 30 °C range. This suggests broader thermal tolerance in the Cambodian populations studied compared to some reports from other regions such as north Queensland in Australia [24], Misiones in Argentina [47] and Recife in Brazil [48]. These variations in optimal survival temperatures may reflect local adaptations to regional climate conditions and differences between mosquito strains [37].

### Adult longevity and vector competence

Vectorial capacity is a measure of the potential of a mosquito population to transmit a pathogen and depends on several biological parameters, including vector-host ratio, mosquito biting rate on humans, daily survival rate, infectiousness of the mosquito to the vertebrate host, susceptibility of the vertebrate host to the virus, extrinsic incubation period and vertebrate host infectious period [50]. Female longevity is a crucial determinant for pathogen transmission, as an extended lifespan increases the probability of multiple blood meals, thereby enhancing the chances of acquiring and transmitting pathogens throughout successive gonotrophic cycles. In our study, the optimal temperature for *Ae. aegypti* and *Ae. albopictus* females’ longevity was between 27.1 and 24.5 °C, respectively. These temperatures are broadly consistent with previous studies, which reported better *Ae. aegypti* longevity between 22 and 28 °C [21] and optimal survival of *Ae. albopictus* adults near 24 °C under controlled conditions [16].

In our study, high blood-feeding rates and high number of eggs laid were recorded at temperatures between 25 and 30 °C for both species. This temperature range is consistent with previous findings, which identified 26 °C to 29 °C as optimal for blood-feeding in *Ae. albopictus*, even in temperate regions such as St. Louis, Missouri, USA [51], with feeding rates declining at temperatures lower or higher than this optimal range. In addition, our results align with previous findings, showing no blood-feeding at 10 °C and limited blood-feeding at 15 °C for both *Ae. aegypti* and *Ae. albopictus* [13, 52], while significantly higher egg production was observed between 25 °C and 30 °C for *Ae. albopictus* [15, 52]. The temperature range of 25–30 °C likely supports optimal mosquito reproduction and population growth, increasing the risk of disease transmission. This contributes to greater vectorial capacity, which is essential for vectorial competence, as mosquitoes that live longer have more opportunities to deliver more infectious bites throughout their lifespan [13, 53–55]. This aligns with global epidemiological patterns, where dengue and other arboviruses are highly endemic in tropical and subtropical regions [56, 57]. These conditions are conducive to higher mosquito densities and increased virus replication rates. Consequently, at temperatures between 25 °C and 28 °C, the extrinsic incubation period of the dengue virus in mosquitoes is shorter, enhancing transmission efficiency [56–58]. The combination of high longevity, frequent blood-feeding and favorable temperature conditions enhances its ability to sustain arbovirus transmission cycles.

Wing length of mosquitoes is often used as a proxy for overall body size [59]. The wing length is negatively correlated with temperature, where larger body sizes are observed at lower temperatures. The large size of the adults is influenced by a prolonged larval stage at colder temperatures, allowing them to accumulate more nutritional reserves [59–62]. Although the wing length is positively associated with fecundity[64], we found that at lower temperatures (15–20 °C), both *Ae. aegypti* and *Ae. albopictus* had larger body sizes yet showed low or no egg production, while higher fecundity occurred at 25–30 °C despite smaller body size. This suggests that temperature influences reproduction through physiological processes, maybe more than the size alone [64]. A larger blood meal may also increase the mosquito’s reproductive output by providing sufficient nutrients for egg development, leading to higher population densities and greater potential for disease transmission in temperate regions [65]. However, to compensate, small mosquitoes exhibit increased feeding frequency to meet their metabolic demands compared with larger individuals [65]. This consequence could influence dengue transmission, as mosquitoes make contact with multiple hosts in a shorter time frame. However, warmer temperatures produce smaller mosquitoes, with a higher risk of dengue transmission due to an increased human biting rate provided by the Ross-Macdonald model [66].

The lowest egg production observed for *Ae. albopictus* was likely attributed to high adult mortality during a 24-h starvation period before blood-feeding, which led to a decrease in the number of mosquitoes available for oviposition. This may underestimate the reproductive potential of the species under certain temperature conditions. Consequently, we found that at 30 °C conditions, *Ae. albopictus* females could lay more than a hundred eggs. In addition, the type of host on which mosquitoes feed can influence their reproductive output and life history traits [67]. We used a single host type (mouse), which may not fully represent the range of physiological responses associated with the natural host preferences of each species. Previous studies have shown that feeding on different vertebrate hosts can result in variations in fecundity, longevity and even vector competence [68, 69]. Finally, the quantity of blood per individual can also influence the egg production because of species-specific differences in physiological efficiency in processing blood as a nutritional resource [63]. These factors should be further researched in future studies to provide a more comprehensive understanding of mosquito reproductive ecology.

### Climate change adaptation

The survival of immature stages and female longevity were highest at 20 °C for *Ae albopictus* and at 25 °C for *Ae aegypti*. *Aedes albopictus*, originating from the forests of Southeast Asia [7, 8], is ecologically adapted to forested habitats, where the dense canopy and vegetation create a cooler and more stable microclimate, buffering temperature extremes. The cooler temperatures in these forested habitats provide the origin site for *Ae. albopictus* in temperate climates and offer optimal conditions for survival. This suggests that *Ae. albopictus* may be better adapted to temperate conditions and spread to colder regions [12, 70]. Due to its greater tolerance for cooler temperatures, *Ae. albopictus* has successfully expanded its spatial distribution into temperate regions, including parts of Europe and North America [71], where it has established stable populations and poses an increasing risk for arbovirus transmission of ZIKV, CHIKV and DENV in Croatia, France, Italy and Spain [72–77].

Conversely, *Ae. aegypti* exhibits a higher survival rate and faster development under warm conditions. We found that *Ae. aegypti* larvae had higher survival rates and developed faster than *Ae. albopictus*, significantly so at 30 and 35 °C. Given its strong association with urban environments and its ability to exploit artificial breeding sites, *Ae. aegypti* is expanding its geographic range as global temperatures rise because of climate change [54]. *Aedes aegypti* thrives in densely populated areas where human activity provides an abundance of water storage containers and microhabitats that facilitate its breeding [11, 55]. However, *Ae. aegypti* is well adapted primarily to tropical and subtropical regions [18, 54], the rise of urban heat and increased temperatures due to human infrastructure, which may further support its expansion into regions that were previously unsuitable [54]. We also found that *Ae. aegypti* can rapidly acclimate to environmental changes through shifts in life history traits, such as shorter development times, increased reproductive output (produce more eggs) and altered feeding behavior. These factors not only enhance its ability to survive in fluctuating climatic conditions but may also increase its vectorial capacity for arboviruses such as dengue, Zika and chikungunya, which could increase its role as a vector in regions where these infections are most prevalent. As climate change progresses, there will be a growing concern that *Ae. aegypti* will establish stable populations in new areas, particularly in regions undergoing rapid urbanization and temperature shifts. Recent findings suggest that while both *Ae. aegypti* and *Ae. albopictus* are expanding their geographic ranges, *Ae. albopictus* demonstrates greater invasiveness because of the increasing temperature in the temperate areas [20]. Climatic and ecological shifts may differentially influence the distribution and dominance of these two species depending on regional conditions.

### Prediction and modeling

Awaiting the development of an efficient and successful vaccine, the most popular method for preventing dengue transmission is still vector control [78]. Given the increasing global temperatures due to climate change, refining predictive models by integrating region- and species-specific biological parameters is essential for effective public health planning. The mathematical and statistical models can help anticipate shifts in vector populations and identify new high-risk areas for dengue outbreaks [79–81]. Some predictive models evaluated the future evolution of *Ae. aegypti* and *Ae. albopictus* distribution in regions like Southeast Asia [19]. However, the reliability of these projections heavily depends on the accuracy and comprehensiveness of the data and models used. This lack of standardization can lead to varying predictions, making it challenging to draw definitive conclusions about the future dynamics of these vector species [19, 82]. Incorporating such a full biological database allows for the calibration and validation of predictive models, thereby improving their accuracy, particularly regarding how mosquito vectors respond to temperature extremes, researchers can significantly improve the predictive power of epidemiological models, leading to more effective public health interventions and reducing the global burden of dengue.

## Conclusions

Our study highlights the significant influence of temperature on the biology, ecology and vectorial capacity of the main dengue vector species, *Ae. aegypti* and *Ae. albopictus*. Elevated temperatures are significant as survival drops dramatically at 35 °C of 40 °C; there are increased blood-feeding frequency and potentially enhanced disease transmission, although these effects may be mitigated by reduced adult longevity. The findings emphasize the complex trade-offs between temperature-driven increases in mosquito activity and survival constraints under extreme heat conditions. We found that temperatures between 25 and 30 °C are most favorable for both species in Phnom Penh, reflecting suitable climatic conditions that are crucial for dengue transmission in the country. Longer-lived females at these temperatures can maintain higher levels of infectivity and increase exposure to human populations over extended periods, contributing to the endemicity of dengue in Asia. Although 25–30 °C represents the optimal conditions for mosquito development, this also poses challenges for effective dengue vector control and prevention strategies, as temperature interacts with various ecological and epidemiological factors. Given the projected rise in global temperatures due to climate change, the expansion of *Ae. aegypti* and *Ae. albopictus* into new geographical regions poses an increasing risk for the spread of arboviral diseases. Temperature-dependent predictive models are needed to improve disease surveillance and vector control strategies, and our data can help this necessary approach. Finally, future research should integrate additional environmental factors such as humidity, urbanization and competition between both species to develop more comprehensive approaches to mitigating the impact of climate change on mosquito-borne disease transmission. In addition, understanding the thermal limits and optimal conditions for mosquito development is crucial for improving vector control strategies and epidemiological modeling. Future studies should also further investigate the vector competence of both species under varying temperature regimes to better predict and mitigate the impacts of climate change on dengue transmission.

## Supplementary Information

Additional file 1. Fig. S1. Developmental duration of *Aedes* *aegypti* and *Aedes* *albopictus* immature stages across different temperatures. **a** Developmental duration of larvae for each species. **b** Species-specific differences in larval development duration. **c** Developmental duration of the pupal stage. **d **Species-specific differences in pupal development duration.

Additional file 2. Fig. S2. Longevity of adult *Aedes* *aegypti* and *Aedes* *albopictus* mosquitoes reared at different temperatures. **a** Longevity of female mosquitoes. **b** Species-specific differences in female longevity. **c** Longevity of male mosquitoes. **d** Species-specific differences in male longevity.

Additional file 3. Table S1. Two-way ANOVA results showing the effects of temperature and species and their interaction across the variables including hatching, larval survival, pupal survival and blood-feeding rate, wing length and number of eggs laid by females

## Data Availability

All raw data analyzed in this study are available on public repository figshare with the following doi: 10.6084/m9.figshare.28844291.v2.
