# Supplementary figures and images for: Impact of temperature on survival, development and longevity of Aedes aegypti and Aedes albopictus (Diptera: Culicidae) in Phnom Penh, Cambodia
Source: Parasit Vectors. 2025 Aug 27;18:362. doi: 10.1186/s13071-025-06892-y (PMC12382174; doi:10.1186/s13071-025-06892-y)

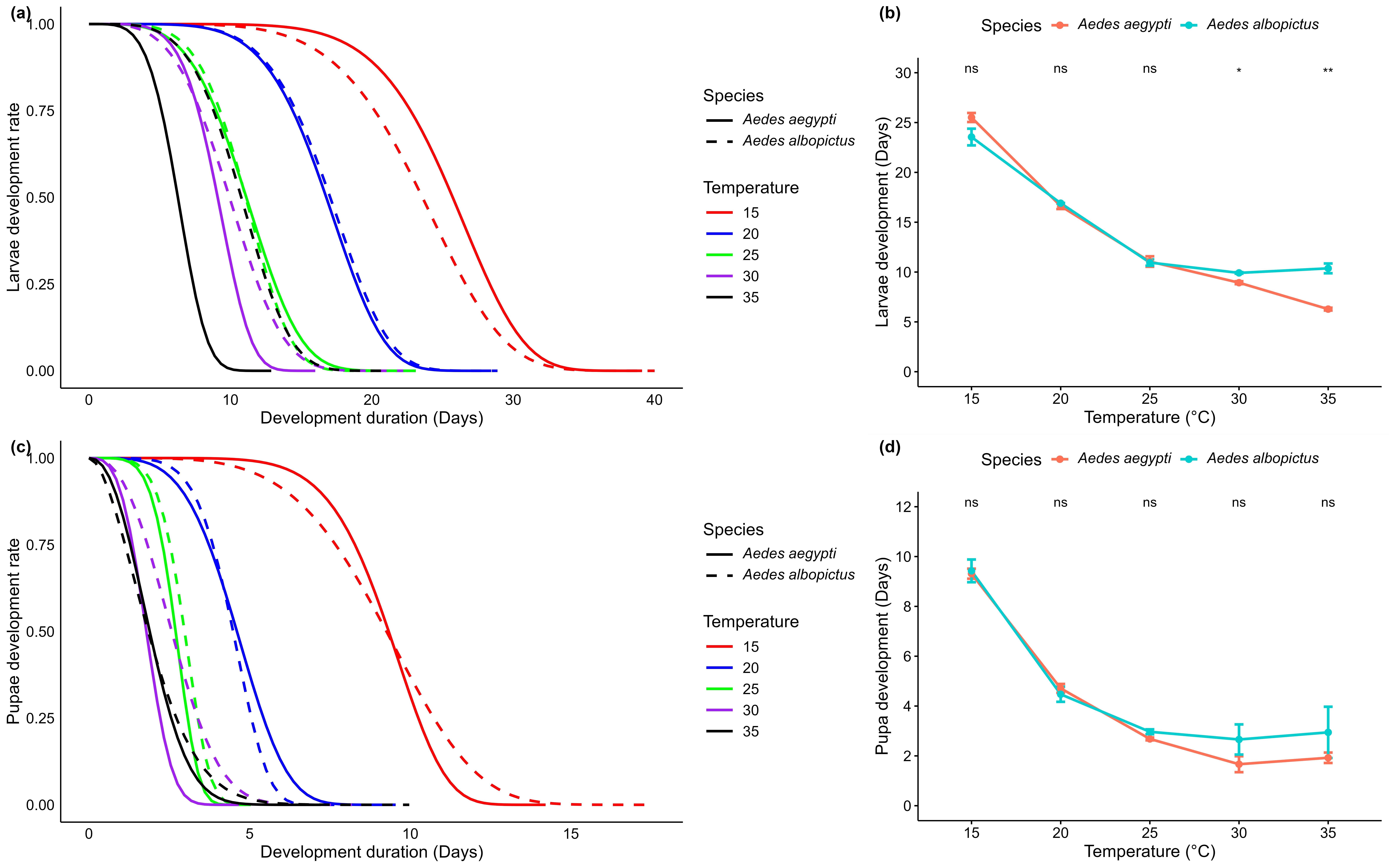

Supplement: Supplementary file 1 — Additional file 1. Fig. S1. Developmental duration of Aedes aegypti and Aedes albopictus immature stages across different temperatures. a Developmental duration of larvae for each species. b Species-specific differences in larval development duration. c Developmental duration of the pupal stage. d Species-specific differences in pupal development duration. [file 13071_2025_6892_MOESM1_ESM.tiff]

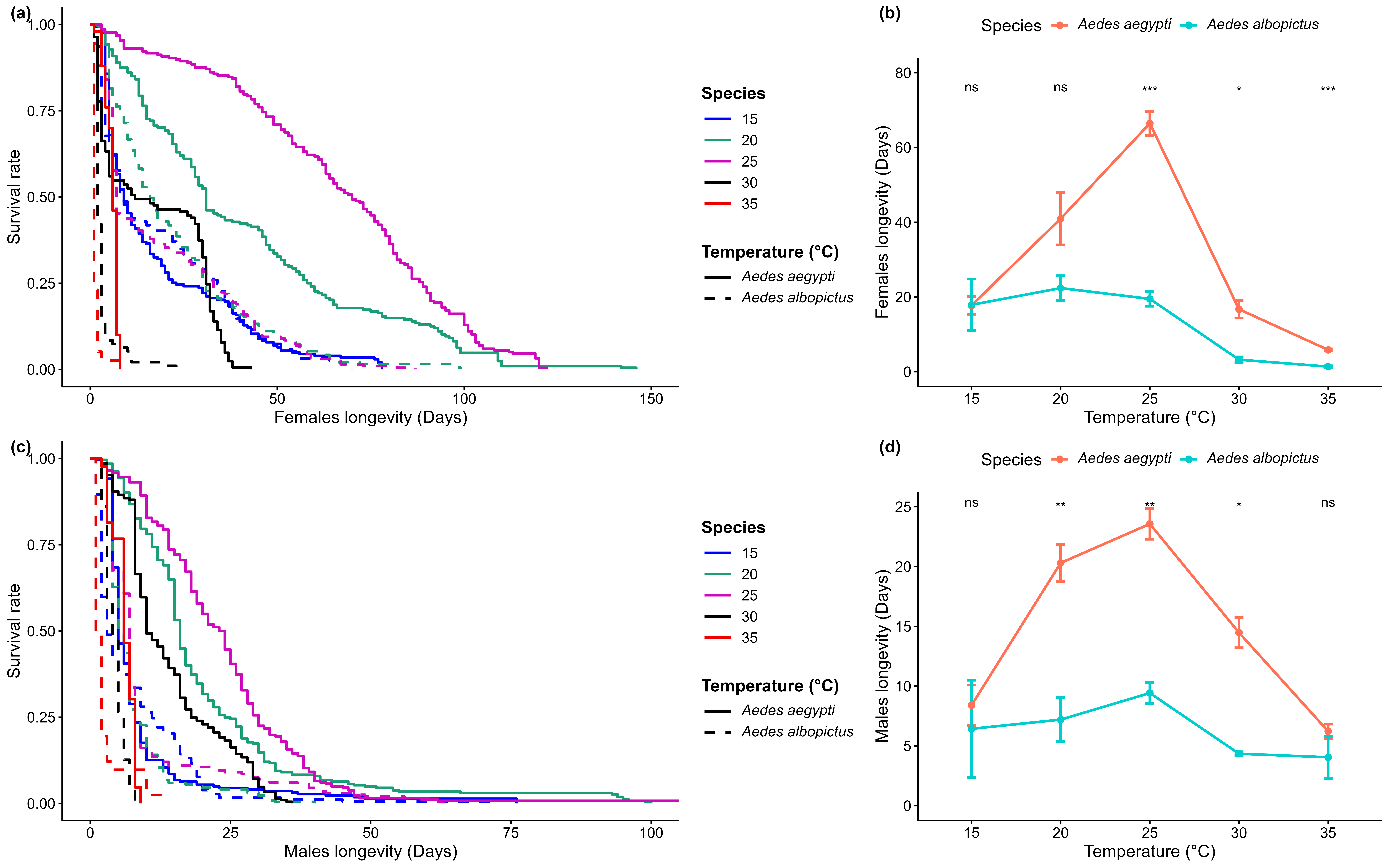

Supplement: Supplementary file 2 — Additional file 2. Fig. S2. Longevity of adult Aedes aegypti and Aedes albopictus mosquitoes reared at different temperatures. a Longevity of female mosquitoes. b Species-specific differences in female longevity. c Longevity of male mosquitoes. d Species-specific differences in male longevity. [file 13071_2025_6892_MOESM2_ESM.tiff]
