# Supplementary material for: Impact of temperature on survival, development and longevity of Aedes aegypti and Aedes albopictus (Diptera: Culicidae) in Phnom Penh, Cambodia
Source: Parasit Vectors. 2025 Aug 27;18:362. doi: 10.1186/s13071-025-06892-y (PMC12382174; doi:10.1186/s13071-025-06892-y)
Supplement: Supplementary file 3 — Additional file 3. Table S1. Two-way ANOVA results showing the effects of temperature and species and their interaction across the variables including hatching, larval survival, pupal survival and blood-feeding rate, wing length and number of eggs laid by females [file 13071_2025_6892_MOESM3_ESM.docx]

**Additional file 3: Table S1**. Two-way ANOVA results showing the effect temperature and species, and the interaction of these across the variables including hatching, larval survival, pupal survival and blood feeding rate, wing length and number egg laying by females.

| Sources of variation | df | Mean square | F | p-value |
| --- | --- | --- | --- | --- |
| Hatching rate |  |  |  |  |
| Temperature | 5 | 7857 | 80.65 | <0.0001 |
| Species | 1 | 928 | 9.53 | 0.005 |
| Temperature x Species | 5 | 55 | 0.57 | 0.72 |
| Larval survival rate |  |  |  |  |
| Temperature | 5 | 8816 | 108.08 | <0.0001 |
| Species | 1 | 0 | 0.001 | 0.97 |
| Temperature x Species | 5 | 19 | 0.23 | 0.94 |
| Pupal survival rate |  |  |  |  |
| Temperature | 4 | 240.85 | 3.58 | 0.02 |
| Species | 1 | 13.74 | 0.21 | 0.66 |
| Temperature x Species | 4 | 15.74 | 0.23 | 0.92 |
| Wing length |  |  |  |  |
| Temperature | 4 | 9.77 | 404.35 | <0.0001 |
| Species | 1 | 1.19 | 49.37 | <0.0001 |
| Temperature x Species | 4 | 0.44 | 18.55 | <0.0001 |
| Blood feeding rate |  |  |  |  |
| Temperature | 3 | 8051 | 19.57 | <0.0001 |
| Species | 1 | 142 | 0.34 | 0.56 |
| Temperature x Species | 3 | 645 | 215 | 0.67 |
| Eggs laying |  |  |  |  |
| Temperature | 3 | 24213 | 42.39 | <0.0001 |
| Species | 1 | 28003 | 49.02 | <0.0001 |
| Temperature x Species | 2 | 9684 | 16.95 | <0.0001 |
